# Pleural recurrence 21 years after complete resection of thymic mucoepidermoid carcinoma: a case report

**DOI:** 10.1186/s44215-023-00124-4

**Published:** 2023-12-01

**Authors:** Shoko Hayashi, Keigo Matsushima, Masashi Mikubo, Masataka Tochimoto, Masaaki Ichinoe, Yukitoshi Satoh

**Affiliations:** 1 Department of Thoracic Surgery, Kitasato University School of Medicine, Sagamihara, Kanagawa 252-0374 Japan; 2 Department of Pathology, Kitasato University School of Medicine, Sagamihara, Kanagawa 252-0374 Japan

**Keywords:** Histological grade, Mucoepidermoid carcinoma, Pleural dissemination, Recurrence, Thymic cancer

## Abstract

**Background:**

Thymic mucoepidermoid carcinomas (MECs) are extremely rare malignant neoplasms. We describe a rare case of a MEC with a high histological grade in a patient who survived for more than 20 years after the initial surgery, focusing on the clinical course and recurrence pattern.

**Case presentation:**

A woman underwent surgical resection of a high-grade thymic MEC at another hospital 21 years ago. At the age of 79, she was referred to our hospital with an abnormal opacity incidentally found on her chest radiograph during a health check-up. After the percutaneous biopsy diagnosed thymic MEC, surgical resection was planned based on imaging findings, considering pulmonary metastasis. Intraoperatively, a large tumor and several nodules were detected within the parietal pleura; furthermore, pleural dissemination of the MEC was diagnosed by intraoperative rapid histological evaluation. We completed an exploratory thoracoscopic procedure without performing resection. She did not wish to undergo adjuvant therapy after surgery. Currently, the tumor is growing slowly, but the patient is asymptomatic and is being followed up without treatment intervention.

**Conclusion:**

We encountered a rare case of pleural recurrence 21 years after complete resection of thymic MEC. Whether surgical resection, including volume reduction surgery, should be used as a treatment strategy for thymic carcinoma with dissemination requires further discussion.

## Background

Mucoepidermoid carcinomas (MECs) are typically found in the salivary tissue and other locations, such as the larynx, bronchus, and esophagus. Thymic MECs are extremely rare [1, 2], accounting for less than 2% of thymic cancers [3]. To our knowledge, less than 30 MEC cases have been reported in the medical literature in the English language [4–8]. The cellular morphology of a thymic MEC is similar to that of its salivary gland counterparts, and there are various combinations of squamous (epidermoid) cells, mucus-producing (goblet) cells, and intermediate cells [9]. They are generally histologically sub-classified into low- and high-grade types based on the proportion of the three cellular components, the level of differentiation, and the invasive pattern [3, 10]. The high-grade tumors behave aggressively with a tendency to invade surrounding tissues and develop metastases [10].

A woman had undergone surgical resection of high-grade thymic MEC at another hospital in 1998. She had a pleural recurrence at 21 years postoperatively. The clinical aspects and etiology of the thymic MECs are still uncertain [1, 4, 5]. Hence, we describe this rare case of MECs with a high histological grade in a patient who survived for more than 20 years after the initial surgery, focusing on the clinical course and recurrence pattern.

### Case presentation

**Fig. 1:**
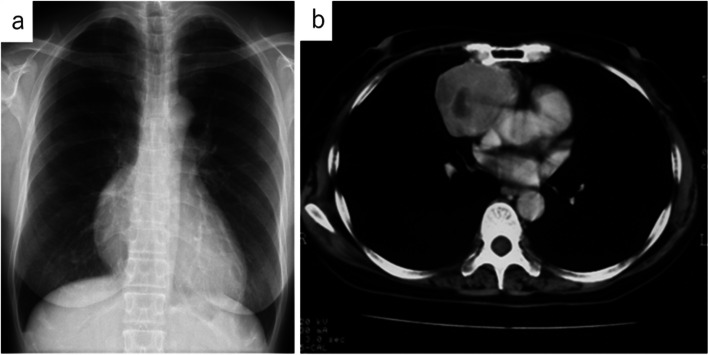
**a** Chest X-ray before the initial surgery. A solid mass with a clearly defined border can be observed in the right lower hilum, showing a positive silhouette sign at the border of the right atrium. **b** Enhanced chest computed tomography scan. An 80-mm solid mass with a clearly defined rim in the anterior mediastinum is observed

In 1998, a 57-year-old Japanese woman was incidentally identified with an asymptomatic mediastinal mass on her chest radiograph during a regular health check-up (Fig. 1a). The levels of serum tumor markers, such as a carcinoembryonic antigen, carbohydrate antigen 19-9, neuron-specific enolase, squamous cell carcinoma antigen, and cytokeratin 19 fragment, were all within their normal ranges. A chest computed tomography (CT) scan revealed a solid 80-mm mass with an irregular rim in the anterior mediastinum (Fig. 1b). Magnetic resonance imaging showed that the mass had not invaded the adjacent structures, such as the right lung, the superior vena cava, and the pericardium. A cytologic diagnosis of adenosquamous carcinoma with the possibility of a primary thymic tumor was made by transcutaneous fine needle aspiration. Based on the thymic carcinoma diagnosis, the patient underwent extended thymus resection combined with wedge resection of the right middle lobe of the lung and pericardial and regional lymph node dissection. The pericardium was reconstructed using a Gore-TEX® patch (W. L. Gore & Associates, Flagstaff, AZ, USA). The patient’s postoperative course was uneventful. Follow-up chest CT scans were performed at regular intervals throughout the 10-year follow-up period, and the patient remained disease-free for more than 10 years. Her follow-up period was subsequently completed.

**Fig. 2:**
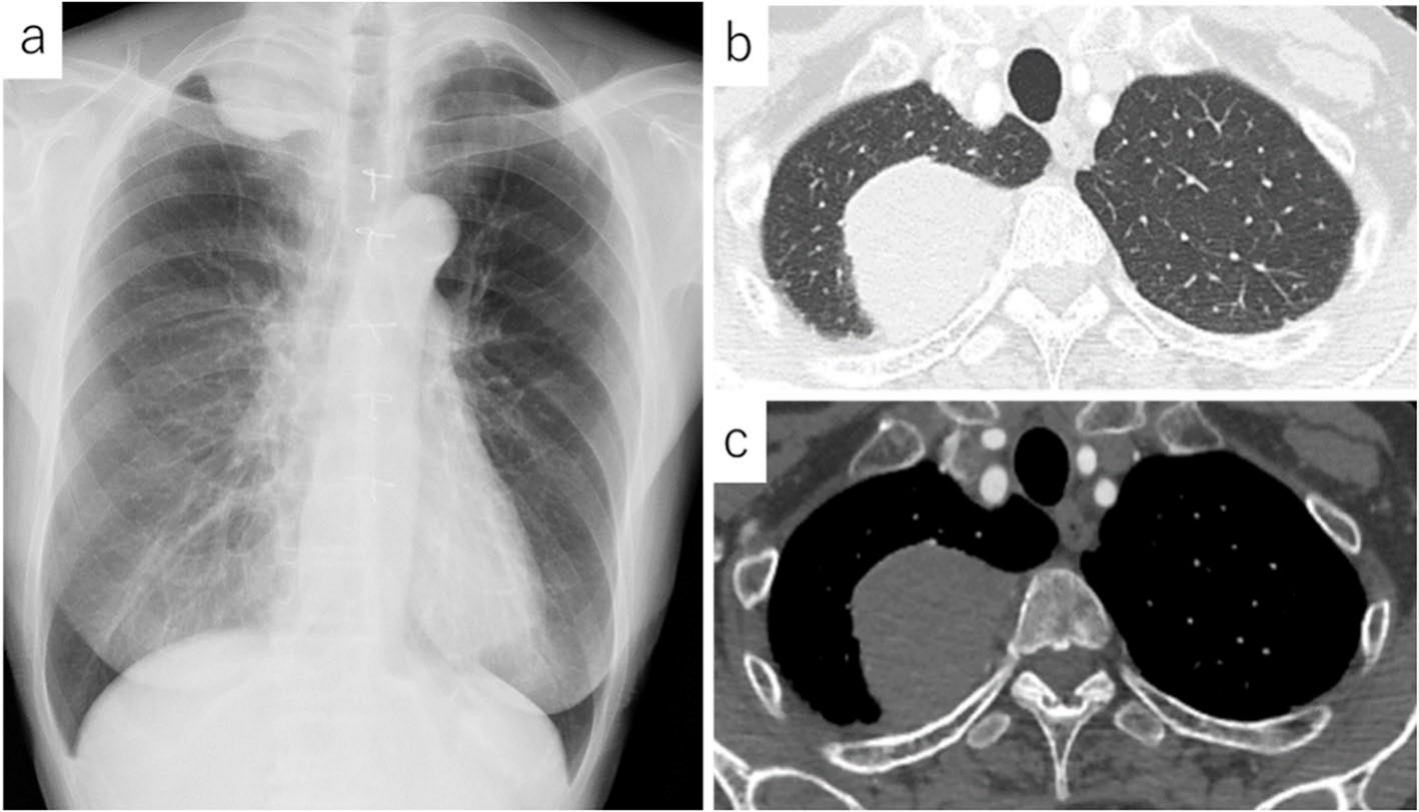
**a** Chest X-ray image 21 years after complete removal. A solid mass with a clearly defined border at the right apex is observed. **b** Enhanced chest computer tomography scan 21 years after complete removal. A 60-mm solid mass with a clearly defined rim in the right upper lobe is observed

**Fig. 3:**
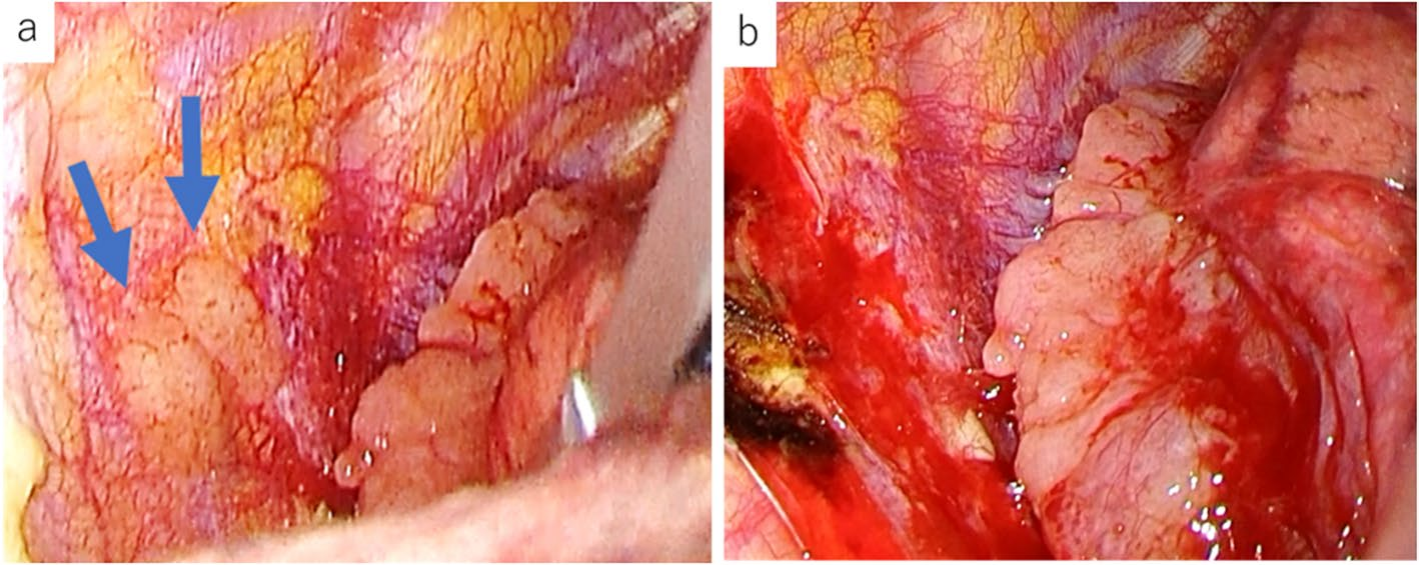
Several disseminated nodules were evident on the parietal pleural surface (arrows) (**a**). The intraoperative findings. A yellow-gray, solid tumor arising from the parietal pleura was invading the right upper lung lobe (**b**)

At the age of 79 years, 21 years after the initial surgery, this patient was referred to us from another hospital for further examination of a mass in the right upper lung field that was found on her chest radiograph during a regular health check-up (Fig. 2a). A CT scan performed at our hospital revealed a solid, 62-mm mass with an irregular rim in the right apical lung field (Fig. 2b). Although both intrapulmonary and extrapulmonary lesions were suggested from these imaging findings, we considered intrapulmonary lesions preoperatively because there was no clear extrapleural sign. The positron emission tomography (PET)/CT revealed fluorodeoxyglucose (FDG) accumulation only in this tumor, with a maximum standard uptake value (SUVmax) of 4.83. Percutaneous aspiration biopsy revealed malignant cells with a mixture of squamous cells and gland formation. We diagnosed pulmonary oligometastasis of the thymic MEC, and a complete resection was planned. During the surgery, a yellow-gray tumor was found, arising from the parietal pleura, that covered the parietal pleura and invaded the right upper lobe of the lung (Fig. 3). There were several nodules on the parietal and visceral pleural surfaces, and pleural dissemination was evident on histology. The significance of surgical procedures, including volume reduction surgery, for thymic carcinoma with dissemination is still debatable. In this case, due to the patient’s resistance to extended resection beyond a lobectomy, we decided not to perform the resection. And she did not wish to undergo postoperative therapy. Two years have passed since the second surgery, and the tumor is growing slowly, but the patient is asymptomatic and is being followed up without treatment intervention.

### Histology

#### Primary lesion at the initial surgery

Macroscopically, the tumor was 70 mm in diameter, rubbery with a clearly defined border, and surrounded by thymic adipose tissue. The cut surface was yellowish-gray, lobulated, and well circumscribed by a thin, fibrous capsule that separated the tumor from the thymic tissue.

**Fig. 4:**
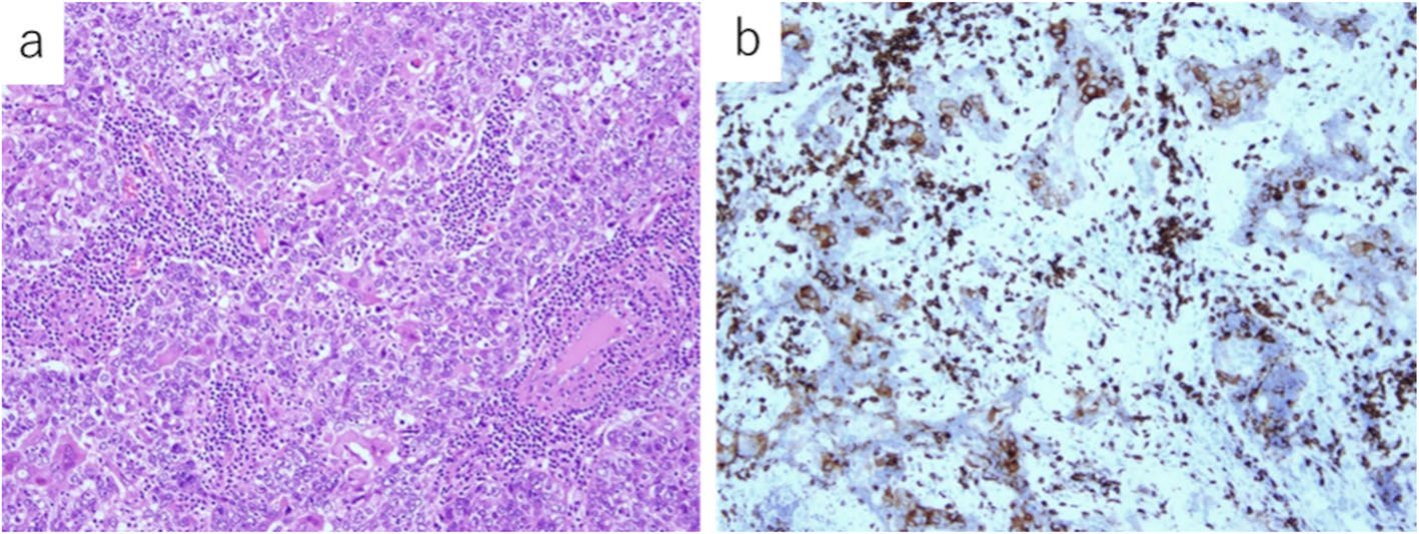
Histological findings of the initial primary lesion. **a** The tumor consists of a mixture of atypical squamous cells and mucus-producing cells with solid cell sheets and no gland formation (hematoxylin and eosin, ×40 objective lens magnification). **b** Immunohistochemically, the tumor cells stain positive for cluster of differentiation (CD) 5 (×100 objective lens magnification)

Microscopically, the tumor consisted of a mixture of atypical squamous and mucus-producing cells with solid cell sheets and no gland formation, with squamous cells comprising approximately 70–80% (Fig. 4a). The nuclei of these cells were large and dysplastic. The squamous components were observed as discrete foci embedded in a cell sheet with relatively abundant eosinophilic cytoplasm and focal intercellular bridges. The goblet-like cells and small spaces stained strongly with periodic acid-Schiff (PAS) and Alcian blue. Immunohistochemically, the tumor cells stained positive for CD5 (Fig. 4b). The tumor had a fibrous capsule and showed no invasion of the surrounding atrophic thymic tissue. No mediastinal lymph node metastasis was observed. The final diagnosis was high-grade thymic MEC due to a predominantly squamous cellular component and strong nuclear atypia.

#### Pleural lesion

**Fig. 5:**
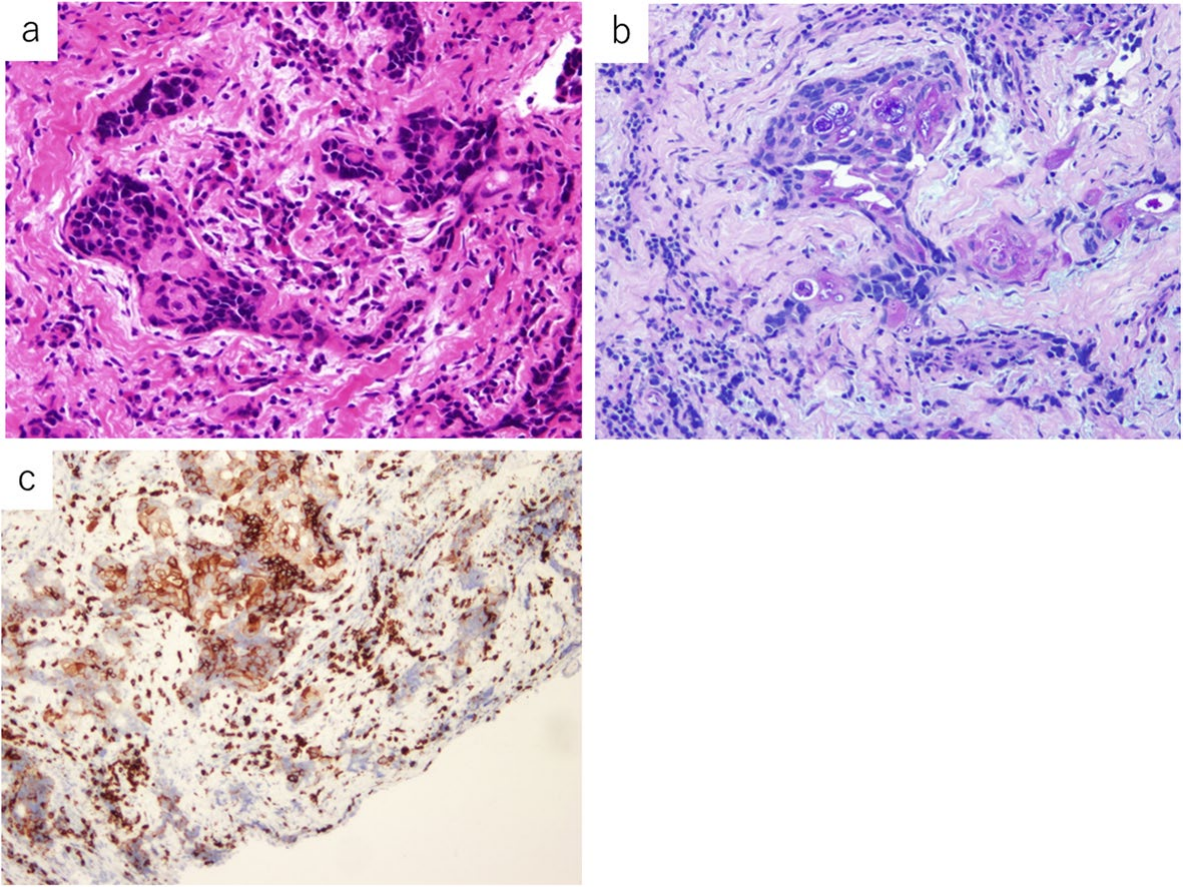
Histological findings of the pleural recurrence. **a** The tumor consists of a mixture of squamous and mucus-producing cells with solid cell sheets and gland formation (hematoxylin and eosin stain, ×400 objective lens magnification). **b** The mucus-producing cells that form the glands are Alcian blue-periodic acid–Schiff stain positive (periodic acid–Schiff stain, ×200 objective lens magnification). **c** Immunohistochemically, the tumor cells stain positive for cluster of differentiation (CD) 5 (×200 objective lens magnification)

Histologically, the tumor was observed to comprise a mixture of squamous and mucus-producing cells with solid cell sheets and gland formation, with squamous cells comprising approximately 70–80% (Fig. 5a). The mucus-producing cells that formed the glands were Alcian blue-PAS positive (Fig. 5b). In the squamous cell component, the tumor cells were focally positive for CD5 (Fig. 5c), p40, and CK5/6, whereas the glandular component was focally positive for CD5 and CK7. Thus, a final diagnosis of dissemination of high-grade thymic MEC was made.

## Discussion

The key points in this case are long-term survival despite high-grade MEC, typically associated with poor prognosis, and the occurrence of a relapse 21 years later. High-grade MECs show poor outcomes with dissemination to the pleura and pericardium, local recurrence, and distant metastasis. Median survival is 9.5 months; 87% of patients died within 1–24 months after surgery [4–7]. On the other hand, the prognosis of low-grade MECs is better [3, 11]; the median survival is 26 months [12]. Our case was very rare because complete resection at the initial presentation resulted in an ultra-long-term survival of 21 years, even though the patient had a high-grade MEC of the thymus. Although there is no consensus on how long MECs should be followed up in terms of prognosis, this case shows that, occasionally, recurrence might occur very late rather than early, even with a high-grade MEC. Once the follow-up has ended, they must still be advised to take regular medical check-ups.

Additionally, whether it was multiple primary metachronous or metastatic recurrences is also controversial. MEC is unlikely to occur in the pleura. The possibility that an extremely rare (0.1–0.2% of lung cancers) [13] primary pulmonary MEC occurred as multiple primary metachronous tumors could not be ruled out completely. However, we consider that it was more likely that thymic MECs recurred in the pleura over a long period of time than that two MECs occurred twice with a 20-year interval between them.

The routes of metastasis of cancer cells into the thoracic cavity include the following: (1) pleural dissemination due to pleural invasion or perforation of the primary tumor, (2) pleural metastasis via the blood or lymphatic route, and (3) extravasation of cancer cells from the circulating blood into the pleural cavity due to hyperpermeability of the vascular membrane. At the time of the initial surgery, the tumor had an intact fibrous capsule with no invasion into the surrounding tissue and was completely resected en bloc. Although no pathologically evident vascular invasion was found at the time of the initial surgery, it is possible that vascular invasion may have occurred despite the limitations of pathology specimen preparation. Both the possibility of dissemination into the thoracic cavity and vascular/lymphatic invasion by preoperative biopsy and the possibility of very small pleural dissemination at the level of a few cells already present before the preoperative biopsy and initial surgery could not be ruled out.

In general, pleural metastatic recurrence of any cancer is most frequent 2–3 years after surgery [14, 15]. Although the mechanism of late recurrence is still unclear, the following two theories have been proposed. Firstly, tumor dormancy theory suggests that recurrence can occur several years or decades after successful primary tumor treatment through surgery or adjuvant therapy [16]. A few cancer cells remain dormant without proliferating, and when they reach a proliferative state at a certain point, they become clinically apparent and can be detected as recurrent lesions [16, 17]. Conversely, the slow-growing tumor theory suggests that tumors with low malignancy and low proliferative potential grow slowly, and recurrent tumors gradually become apparent over a long period of time [18].

The differential diagnosis of mediastinal tumors is often possible based on clinical information, imaging findings, and tumor marker values. Currently, percutaneous needle biopsy should not be recommended if thymic epithelial tumors are suspected and are expected to be resectable [19]. A biopsy is recommended when seminoma or malignant lymphoma is strongly suspected or when medical therapy is administered in unresectable cases [19]. The possibility of biopsy-induced manipulation or dissemination should always be considered.

In this case, for macroscopic complete resection, a disseminated nest resection, including right upper lobectomy and combined chest wall resection, may be necessary. We consider the significance of gross resection in cases of dissemination. Volume reduction is recommended as much as possible, even for stage IV thymomas [19]. Patients with macroscopic resection of all pleural dissemination had a better prognosis than those with incomplete resection [20, 21], with similar results in the meta-analysis [22]. On the other hand, although incomplete resection has not been considered to benefit survival in thymic carcinoma with dissemination, it may still yield a more favorable prognosis after incomplete resection was better compared to non-resection [23]. Due to the small number of cases, it is necessary to discuss them further. Similarly, in non-small-cell lung cancer (NSCLC) with pleural dissemination, it is generally controversial to perform surgery. However, in recent years, several reports have demonstrated the efficacy of local excision of the primary tumor and pleurectomy for pleural dissemination [24–26]. To evaluate the benefit of adding primary tumor resection to standard chemotherapy for NSCLC with pleural dissemination, the JCOG2103 [27] trial has begun, and the results are awaited.

Although the addition of radiation therapy after macroscopic resection for local recurrence is controversial, it may be considered in cases of positive margins or in lesions with such potential. Radiation therapy should be discussed with the radiotherapists considering complication possibilities, including interstitial pneumonia.

## Conclusion

We encountered a rare case of pleural recurrence 21 years after complete resection of thymic MEC. Whether surgical resection, including volume reduction surgery, should be used as a treatment strategy for thymic carcinoma with dissemination, unlike thymoma, requires further discussion.

## Data Availability

The authors have full control of all primary data and agree to allow the journal to review their data if requested.

## Abbreviations

MEC: Mucoepidermoid carcinomas
CK: Cytokeratin
CT: Computed tomography
PAS: Periodic acid-Schiff
CD5: Cluster of differentiation 5
p40: Tumor protein 40
